# 4-Methyl-*N*-(3-methyl­phen­yl)pyridin-2-amine

**DOI:** 10.1107/S1600536811044059

**Published:** 2011-10-29

**Authors:** Zainal Abidin Fairuz, Zaharah Aiyub, Zanariah Abdullah, Seik Weng Ng, Edward R. T. Tiekink

**Affiliations:** a Department of Chemistry, University of Malaya, 50603 Kuala Lumpur, Malaysia; b Chemistry Department, Faculty of Science, King Abdulaziz University, PO Box 80203 Jeddah, Saudi Arabia

## Abstract

The title amine, C_13_H_14_N_2_, is twisted with a dihedral angle between the rings of 60.07 (9)°. The amine N—H group and pyridine N atom are *syn* allowing for the formation of centrosymmetric eight-membered {⋯HNCN}_2_ synthons *via* N—H⋯N hydrogen bonds. The two-mol­ecule aggregates are sustained in the three-dimensional crystal packing *via* C—H⋯π and π–π inter­actions [centroid–centroid distance for pyridyl rings = 3.7535 (12) Å]

## Related literature

For a copper(II) paddle-wheel complex containing the title mol­ecule as a ligand, see: Fairuz *et al.* (2010 ▶).
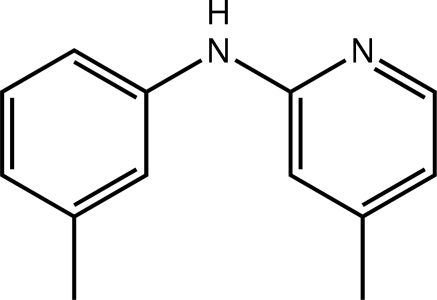


## Experimental

### 

#### Crystal data

C_13_H_14_N_2_
*M* *_r_* = 198.26
Triclinic, 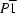

*a* = 7.1802 (9) Å
*b* = 7.6509 (10) Å
*c* = 10.8120 (14) Å
α = 106.957 (2)°
β = 91.859 (2)°
γ = 95.720 (2)°
*V* = 564.12 (13) Å^3^
*Z* = 2
Mo *K*α radiation
μ = 0.07 mm^−1^
*T* = 293 K
0.20 × 0.18 × 0.10 mm

#### Data collection

Bruker SMART APEX diffractometer
Absorption correction: multi-scan (*SADABS*; Sheldrick, 1996 ▶) *T*_min_ = 0.986, *T*_max_ = 0.993
7262 measured reflections
2581 independent reflections
1694 reflections with *I* > 2σ(*I*)
*R*_int_ = 0.029

#### Refinement

*R*[*F* ^2^ > 2σ(*F* ^2^)] = 0.053
*wR*(*F* ^2^) = 0.168
*S* = 1.02
2581 reflections
142 parameters
1 restraint
H atoms treated by a mixture of independent and constrained refinement
Δρ_max_ = 0.24 e Å^−3^
Δρ_min_ = −0.19 e Å^−3^

### 

Data collection: *APEX2* (Bruker, 2009 ▶); cell refinement: *SAINT* (Bruker, 2009 ▶); data reduction: *SAINT*; program(s) used to solve structure: *SHELXS97* (Sheldrick, 2008 ▶); program(s) used to refine structure: *SHELXL97* (Sheldrick, 2008 ▶); molecular graphics: *ORTEP-3* (Farrugia, 1997 ▶) and *DIAMOND* (Brandenburg, 2006 ▶); software used to prepare material for publication: *publCIF* (Westrip, 2010 ▶).

## Supplementary Material

Crystal structure: contains datablock(s) global, I. DOI: 10.1107/S1600536811044059/hg5117sup1.cif

Structure factors: contains datablock(s) I. DOI: 10.1107/S1600536811044059/hg5117Isup2.hkl

Supplementary material file. DOI: 10.1107/S1600536811044059/hg5117Isup3.cml

Additional supplementary materials:  crystallographic information; 3D view; checkCIF report

## Figures and Tables

**Table 1:** Hydrogen-bond geometry (Å, °) *Cg*1 and *Cg*2 are centroids of the N2,C1–C5 and C7–C12 rings, respectively.

*D*—H⋯*A*	*D*—H	H⋯*A*	*D*⋯*A*	*D*—H⋯*A*
N1—H1*n*⋯N2^i^	0.87 (1)	2.12 (1)	2.978 (2)	172 (2)
C6—H6b⋯*Cg*1^ii^	0.96	2.73	3.624 (3)	155
C13—H13b⋯*Cg*2^iii^	0.96	2.74	3.612 (2)	151
C13—H13c⋯*Cg*2^iv^	0.96	2.88	3.642 (2)	137

Symmetry codes: (i) 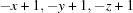; (ii) 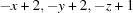; (iii) 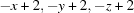; (iv) 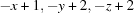.

**Fig. 1.**
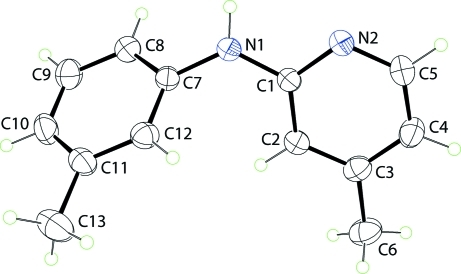
The molecular structure of (I) showing the atom-labelling scheme and displacement ellipsoids at the 35% probability level.

**Fig. 2.**
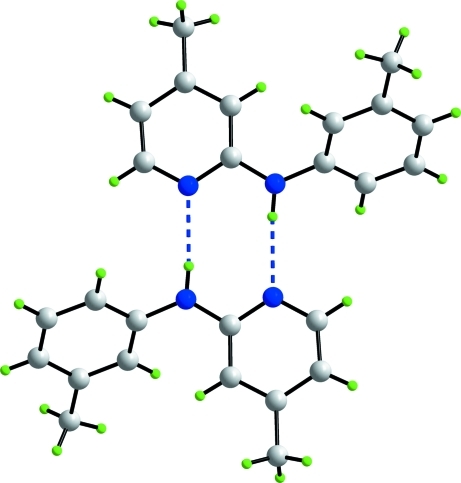
Two molecule aggregates in (I) mediated by N—H···N hydrogen bonding, shown as blue dashed lines.

**Fig. 3.**
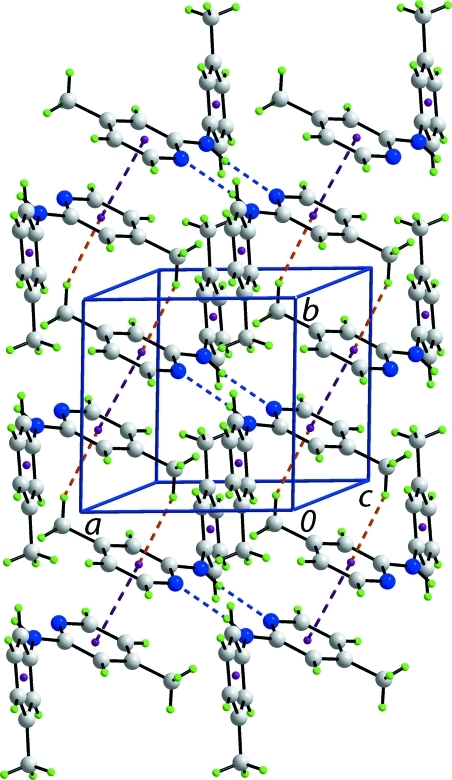
Layers in the ab plane in (I) sustained by N—H···N hydrogen bonding, C—H···π(pyridyl) and π–π interactions shown as blue, orange and purple dashed lines, respectively.

**Fig. 4.**
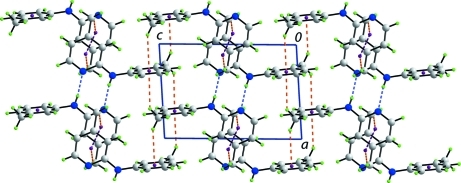
Unit-cell contents for (I) shown in projection down the b axis highlighting the inter-digitation of layers. The N—H···N hydrogen bonding, C—H···π(pyridyl) and π–π interactions are shown as blue, orange and purple dashed lines, respectively.

## supplementary crystallographic information

### Comment

The title amine, (I), has been observed to coordinate Cu^II^ in a paddle-wheel motif (Fairuz *et al.*, 2010). Herein, the crystal and molecular structure of the amine is described.

The dihedral angle between the pyridyl and benzene rings in (I), Fig. 1, is 60.07 (9)°, indicating a twisted conformation. The amine-NH and pyridyl-N atoms are *syn*. This latter feature allows for the formation of centrosymmetric eight-membered {···HNCN}_2_ synthons *via* N—H···N hydrogen bonds, Fig. 2 and Table 1. The two-molecule aggregates are connected into a layer in the *ab* plane *via* C—H···π(pyridyl) interactions, Table 1, and π–π interactions occurring between pyridyl rings [3.7535 (12) Å for symmetry operation 2 - *x*, 1 - *y*, 1 - *z*], Fig. 3. The benzene rings project out of the layers allowing for their inter-digitation along the *c* axis *via* C—H···π interactions, Fig. 4 and Table 1.

### Experimental

2-Chloro-4-methylpyridine (1.0 ml, 1.14 mmol) and *m*-toluidine (1.24 ml, 1.14 mmol) were refluxed for 4 h. The suspension was cooled, taken up in water (15 ml) and then extracted with diethyl ether (3 *x* 10 ml). The organic layer was washed with water (3 *x* 10 ml) and dried over anhydrous sodium sulfate. Evaporation of diethyl ether gave a dark-brown solid and recrystallization from its ethanol solution gave pure colourless crystals.

### Refinement

Hydrogen atoms were placed at calculated positions (C—H 0.95 Å) and were treated as riding on their parent carbon atoms, with *U*(H) set to 1.2–1.5*U*_eq_(C). The N-bound H-atom was located in a difference Fourier map and was refined with N—H = 0.86±0.01 Å, and with unconstrained *U*_iso_.

### Crystal data

**Table:**

C_13_H_14_N_2_	*Z* = 2
*M**_r_* = 198.26	*F*(000) = 212
Triclinic, *P*1	*D*_x_ = 1.167 Mg m^−^^3^
Hall symbol: -P 1	Mo *K*α radiation, λ = 0.71073 Å
*a* = 7.1802 (9) Å	Cell parameters from 1537 reflections
*b* = 7.6509 (10) Å	θ = 2.8–24.2°
*c* = 10.8120 (14) Å	µ = 0.07 mm^−^^1^
α = 106.957 (2)°	*T* = 293 K
β = 91.859 (2)°	Prism, colourless
γ = 95.720 (2)°	0.20 × 0.18 × 0.10 mm
*V* = 564.12 (13) Å^3^	

### Data collection

**Table:**

Bruker SMART APEX diffractometer	2581 independent reflections
Radiation source: fine-focus sealed tube	1694 reflections with *I* > 2σ(*I*)
graphite	*R*_int_ = 0.029
ω scans	θ_max_ = 27.5°, θ_min_ = 2.0°
Absorption correction: multi-scan (*SADABS*; Sheldrick, 1996)	*h* = −8→9
*T*_min_ = 0.986, *T*_max_ = 0.993	*k* = −9→9
7262 measured reflections	*l* = −12→14

### Refinement

**Table:**

Refinement on *F*^2^	Primary atom site location: structure-invariant direct methods
Least-squares matrix: full	Secondary atom site location: difference Fourier map
*R*[*F*^2^ > 2σ(*F*^2^)] = 0.053	Hydrogen site location: inferred from neighbouring sites
*wR*(*F*^2^) = 0.168	H atoms treated by a mixture of independent and constrained refinement
*S* = 1.02	*w* = 1/[σ^2^(*F*_o_^2^) + (0.0822*P*)^2^ + 0.1088*P*] where *P* = (*F*_o_^2^ + 2*F*_c_^2^)/3
2581 reflections	(Δ/σ)_max_ < 0.001
142 parameters	Δρ_max_ = 0.24 e Å^−^^3^
1 restraint	Δρ_min_ = −0.19 e Å^−^^3^

### Fractional atomic coordinates and isotropic or equivalent isotropic displacement parameters (Å^2^)

**Table:**

	*x*	*y*	*z*	*U*_iso_*/*U*_eq_	
N1	0.6599 (2)	0.6400 (2)	0.64600 (14)	0.0512 (4)	
H1n	0.561 (2)	0.563 (2)	0.6173 (18)	0.061 (6)*	
N2	0.7026 (2)	0.6004 (2)	0.43224 (13)	0.0445 (4)	
C1	0.7759 (2)	0.6687 (2)	0.55430 (15)	0.0384 (4)	
C2	0.9589 (2)	0.7569 (2)	0.58388 (16)	0.0424 (4)	
H2	1.0056	0.8030	0.6698	0.051*	
C3	1.0702 (3)	0.7759 (2)	0.48638 (18)	0.0452 (4)	
C4	0.9924 (3)	0.7063 (3)	0.35997 (18)	0.0537 (5)	
H4	1.0613	0.7182	0.2909	0.064*	
C5	0.8129 (3)	0.6199 (3)	0.33856 (17)	0.0530 (5)	
H5	0.7643	0.5716	0.2532	0.064*	
C6	1.2683 (3)	0.8666 (3)	0.5154 (2)	0.0616 (6)	
H6A	1.3222	0.8406	0.5897	0.092*	
H6B	1.2691	0.9972	0.5331	0.092*	
H6C	1.3405	0.8202	0.4422	0.092*	
C7	0.6915 (2)	0.7151 (2)	0.78132 (15)	0.0408 (4)	
C8	0.6745 (3)	0.6003 (3)	0.85864 (17)	0.0483 (5)	
H8	0.6491	0.4739	0.8210	0.058*	
C9	0.6951 (3)	0.6721 (3)	0.99134 (19)	0.0592 (5)	
H9	0.6829	0.5941	1.0431	0.071*	
C10	0.7336 (3)	0.8593 (3)	1.04803 (18)	0.0580 (5)	
H10	0.7490	0.9063	1.1378	0.070*	
C11	0.7494 (2)	0.9777 (3)	0.97289 (18)	0.0488 (5)	
C12	0.7282 (2)	0.9038 (2)	0.83984 (17)	0.0460 (4)	
H12	0.7386	0.9821	0.7881	0.055*	
C13	0.7845 (3)	1.1821 (3)	1.0352 (2)	0.0708 (7)	
H13A	0.7804	1.2436	0.9696	0.106*	
H13B	0.9056	1.2125	1.0811	0.106*	
H13C	0.6897	1.2207	1.0947	0.106*	

### Atomic displacement parameters (Å^2^)

**Table:**

	*U*^11^	*U*^22^	*U*^33^	*U*^12^	*U*^13^	*U*^23^
N1	0.0469 (9)	0.0642 (10)	0.0337 (8)	−0.0178 (8)	−0.0010 (7)	0.0089 (7)
N2	0.0478 (9)	0.0479 (8)	0.0344 (8)	−0.0035 (6)	−0.0010 (6)	0.0105 (6)
C1	0.0425 (9)	0.0371 (8)	0.0336 (9)	−0.0011 (7)	0.0002 (7)	0.0094 (6)
C2	0.0412 (10)	0.0439 (9)	0.0389 (9)	−0.0001 (7)	−0.0010 (7)	0.0094 (7)
C3	0.0429 (10)	0.0388 (8)	0.0555 (11)	0.0066 (7)	0.0092 (8)	0.0151 (8)
C4	0.0577 (12)	0.0583 (11)	0.0470 (11)	0.0047 (9)	0.0167 (9)	0.0178 (9)
C5	0.0619 (13)	0.0587 (11)	0.0353 (9)	0.0010 (9)	0.0038 (9)	0.0109 (8)
C6	0.0429 (11)	0.0623 (12)	0.0786 (15)	−0.0001 (9)	0.0112 (10)	0.0204 (11)
C7	0.0333 (9)	0.0509 (10)	0.0349 (9)	−0.0009 (7)	0.0007 (7)	0.0094 (7)
C8	0.0512 (11)	0.0478 (10)	0.0446 (10)	−0.0004 (8)	0.0034 (8)	0.0135 (8)
C9	0.0670 (14)	0.0693 (13)	0.0443 (11)	0.0016 (10)	0.0031 (9)	0.0237 (10)
C10	0.0566 (12)	0.0755 (14)	0.0339 (9)	0.0037 (10)	0.0018 (8)	0.0050 (9)
C11	0.0356 (9)	0.0530 (10)	0.0483 (11)	0.0067 (8)	−0.0004 (8)	0.0001 (8)
C12	0.0434 (10)	0.0478 (10)	0.0466 (10)	0.0020 (7)	−0.0014 (8)	0.0153 (8)
C13	0.0576 (13)	0.0582 (12)	0.0774 (15)	0.0090 (10)	−0.0050 (11)	−0.0096 (11)

### Geometric parameters (Å, °)

**Table:**

N1—C1	1.367 (2)	C6—H6C	0.9600
N1—C7	1.409 (2)	C7—C8	1.378 (2)
N1—H1n	0.865 (10)	C7—C12	1.391 (2)
N2—C1	1.338 (2)	C8—C9	1.376 (3)
N2—C5	1.339 (2)	C8—H8	0.9300
C1—C2	1.398 (2)	C9—C10	1.378 (3)
C2—C3	1.375 (2)	C9—H9	0.9300
C2—H2	0.9300	C10—C11	1.382 (3)
C3—C4	1.389 (3)	C10—H10	0.9300
C3—C6	1.500 (3)	C11—C12	1.381 (2)
C4—C5	1.368 (3)	C11—C13	1.503 (3)
C4—H4	0.9300	C12—H12	0.9300
C5—H5	0.9300	C13—H13A	0.9600
C6—H6A	0.9600	C13—H13B	0.9600
C6—H6B	0.9600	C13—H13C	0.9600
			
C1—N1—C7	126.61 (15)	C8—C7—C12	118.87 (16)
C1—N1—H1n	115.9 (14)	C8—C7—N1	119.37 (16)
C7—N1—H1n	117.4 (14)	C12—C7—N1	121.64 (16)
C1—N2—C5	116.93 (15)	C7—C8—C9	120.19 (17)
N2—C1—N1	114.66 (15)	C7—C8—H8	119.9
N2—C1—C2	122.02 (15)	C9—C8—H8	119.9
N1—C1—C2	123.28 (15)	C8—C9—C10	120.34 (19)
C3—C2—C1	120.26 (16)	C8—C9—H9	119.8
C3—C2—H2	119.9	C10—C9—H9	119.8
C1—C2—H2	119.9	C9—C10—C11	120.69 (18)
C2—C3—C4	117.35 (17)	C9—C10—H10	119.7
C2—C3—C6	121.26 (18)	C11—C10—H10	119.7
C4—C3—C6	121.38 (17)	C12—C11—C10	118.37 (17)
C5—C4—C3	119.02 (17)	C12—C11—C13	121.09 (19)
C5—C4—H4	120.5	C10—C11—C13	120.52 (18)
C3—C4—H4	120.5	C11—C12—C7	121.52 (17)
N2—C5—C4	124.40 (17)	C11—C12—H12	119.2
N2—C5—H5	117.8	C7—C12—H12	119.2
C4—C5—H5	117.8	C11—C13—H13A	109.5
C3—C6—H6A	109.5	C11—C13—H13B	109.5
C3—C6—H6B	109.5	H13A—C13—H13B	109.5
H6A—C6—H6B	109.5	C11—C13—H13C	109.5
C3—C6—H6C	109.5	H13A—C13—H13C	109.5
H6A—C6—H6C	109.5	H13B—C13—H13C	109.5
H6B—C6—H6C	109.5		
			
C5—N2—C1—N1	−177.95 (16)	C1—N1—C7—C8	−129.1 (2)
C5—N2—C1—C2	0.1 (2)	C1—N1—C7—C12	54.9 (3)
C7—N1—C1—N2	−171.86 (17)	C12—C7—C8—C9	−0.5 (3)
C7—N1—C1—C2	10.1 (3)	N1—C7—C8—C9	−176.57 (17)
N2—C1—C2—C3	0.0 (3)	C7—C8—C9—C10	−0.3 (3)
N1—C1—C2—C3	177.81 (16)	C8—C9—C10—C11	0.9 (3)
C1—C2—C3—C4	0.7 (3)	C9—C10—C11—C12	−0.8 (3)
C1—C2—C3—C6	−178.78 (16)	C9—C10—C11—C13	177.77 (19)
C2—C3—C4—C5	−1.4 (3)	C10—C11—C12—C7	0.0 (3)
C6—C3—C4—C5	178.08 (18)	C13—C11—C12—C7	−178.52 (17)
C1—N2—C5—C4	−0.9 (3)	C8—C7—C12—C11	0.6 (3)
C3—C4—C5—N2	1.6 (3)	N1—C7—C12—C11	176.60 (16)

### Hydrogen-bond geometry (Å, °)

**Table:**

Cg1 and Cg2 are centroids of the N2,C1–C5 and C7–C12 rings, respectively.

**Table:**

*D*—H···*A*	*D*—H	H···*A*	*D*···*A*	*D*—H···*A*
N1—H1n···N2^i^	0.87 (1)	2.12 (1)	2.978 (2)	172.(2)
C6—H6b···Cg1^ii^	0.96	2.73	3.624 (3)	155
C13—H13b···Cg2^iii^	0.96	2.74	3.612 (2)	151
C13—H13c···Cg2^iv^	0.96	2.88	3.642 (2)	137

Symmetry codes: (i) −*x*+1, −*y*+1, −*z*+1; (ii) −*x*+2, −*y*+2, −*z*+1; (iii) −*x*+2, −*y*+2, −*z*+2; (iv) −*x*+1, −*y*+2, −*z*+2.
